# Serum albumin-to-creatinine ratio as a novel and cost-effective biomarker for silent cerebral infarction: a retrospective cohort study

**DOI:** 10.3389/fneur.2025.1633402

**Published:** 2025-08-22

**Authors:** Mustafa Mazıcan, Ismail Karluka

**Affiliations:** Adana Dr. Turgut Noyan Application and Research Center, Department of Interventional Radiology, Division of Cerebrovascular Disease and Stroke, Başkent University, Adana, Türkiye

**Keywords:** serum albumin to creatinine ratio, cost-effective biomarker, silent cerebral infarction, subclinical cerebrovascular disease, biomarker-based risk stratification

## Abstract

**Background/objectives:**

Silent cerebral infarction (SCI) is a common but underrecognized condition characterized by asymptomatic cerebral lesions detected via magnetic resonance imaging (MRI). While traditional vascular risk factors contribute to its pathogenesis, novel biomarkers may enhance risk stratification. The serum albumin to creatinine ratio (sACR) reflects nutritional status and renal function—two factors closely linked to vascular health. This study aimed to investigate the association between sACR and the presence of SCI, as well as its potential prognostic value for long-term outcomes.

**Methods:**

We retrospectively analyzed 272 consecutive patients who visited the neurology outpatient clinic and underwent brain MRI for any reason, provided they met the study’s inclusion criteria. Patients with a history of cerebrovascular disease or other exclusion criteria were not included. Baseline demographic and laboratory data, including sACR, were collected. SCI was identified using standardized imaging criteria. Adverse outcomes, including cerebrovascular events and atrial fibrillation/flutter, were assessed over a median follow-up period of 37 months.

**Results:**

Patients with SCI had significantly 24 lower sACR levels compared to those without SCI. In multivariable logistic regression analysis, each 1-point decrease in sACR was independently associated with a 1.790-fold increase in the odds of SCI (95% CI: 1.384–2.315, *p* < 0.001). ROC curve analysis yielded a modest discriminatory ability for sACR in predicting SCI (AUC = 0.674; cut-off: 5.13). Kaplan–Meier survival analysis showed a trend toward higher long-term outcome in the low sACR group, but this did not reach statistical significance (log-rank *p* = 0.088).

**Conclusion:**

Lower sACR levels are independently associated with the presence of SCI. While the association between sACR and long-term outcomes requires further investigation, sACR may serve as a cost-effective biomarker for assessing subclinical cerebrovascular risk. Future prospective studies are needed to validate these findings and clarify the clinical utility of sACR-guided risk stratification.

## 1 Introduction

Silent cerebral infarction (SCI) is a prevalent yet often underdiagnosed condition characterized by asymptomatic cerebral lesions detected through magnetic resonance imaging (MRI). Despite the absence of clinical symptoms, SCI has significant prognostic implications, being associated with cognitive decline, stroke recurrence, and increased cardiovascular morbidity (1–3). Identifying modifiable risk factors early is critical for its prevention and management.

While traditional vascular risk factors—such as hypertension, diabetes, and hyperlipidemia—have been extensively studied concerning SCI, recent research has shifted toward identifying novel biomarkers with additional predictive value. Among these, markers of renal function and protein metabolism have emerged as relevant indicators of vascular health (4–6). One such marker, the serum albumin-to-creatinine ratio (sACR), reflects both nutritional status and renal function. Although sACR has gained attention in the context of cardiac ischemia, heart failure, and sepsis (7–9), its relevance to cerebrovascular disease, and particularly to SCI, remains unexplored. Prior studies have focused mainly on the urinary albumin-to-creatinine ratio (uACR) with cerebral atherosclerosis and stroke risk (10, 11).

Recent investigations have underscored the interplay of systemic inflammation, oxidative stress, and endothelial dysfunction in the pathogenesis of cerebrovascular disease, including SCI. Serum albumin maintains oncotic pressure and exerts anti-inflammatory, antioxidant, and endothelial-protective effects (12, 13). Hypoalbuminemia has been independently linked to the higher incidence of ischemic heart disease, heart failure, atrial fibrillation, stroke, and venous thromboembolism—even after adjustment for traditional cardiovascular risk factors (12)—and correlates with elevated soluble adhesion molecules as markers of endothelial activation (14). Albumin preserves vascular integrity by scavenging reactive oxygen and nitrogen species and stabilizing the endothelial glycocalyx (13). In addition to conventional vascular risk factors, serum biomarkers such as interleukin-6 (IL-6) and C-reactive protein (CRP) have been studied about silent brain infarction (SCI), reflecting systemic inflammation. Furthermore, oxidative stress and endothelial dysfunction have also been proposed as key mechanisms underlying silent brain injury (15). However, the potential utility of integrated nutritional and renal biomarkers like the serum albumin-to-creatinine ratio (sACR) remains underexplored.

SCI is thought to arise from chronic small vessel damage, endothelial dysfunction, and subclinical atherosclerosis—processes influenced by systemic inflammation and renal insufficiency (15). Serum albumin, a marker of nutritional and anti-inflammatory status, and serum creatinine, an indicator of renal and vascular health, provide a composite picture of systemic vascular vulnerability. Thus, the sACR may represent an integrated biomarker reflecting these interrelated pathological processes.

Emerging evidence highlights that low serum albumin levels are independently linked to increased risks of ischemic stroke and vascular events (16). In contrast, elevated serum creatinine is associated with arterial stiffness, inflammation, and endothelial dysfunction—all relevant to SCI pathophysiology (17). Similarly, indices like the fibrinogen-to-albumin ratio (FAR) have shown associations with stroke risk in small vessel disease (16), supporting the prognostic relevance of albumin-based markers in cerebrovascular pathology.

Taken together, sACR may serve as a practical, cost-effective biomarker for early risk stratification of cerebrovascular vulnerability, integrating key systemic processes involved in SCI development.

This study aims to evaluate, for the first time, the association between sACR and SCI, and to explore the potential prognostic implications of baseline SCI concerning long-term outcomes (stroke, atrial fibrillation/flutter). As a retrospective cross-sectional analysis, the study does not imply causality. Instead, it seeks to contribute to preventive strategies by examining a novel, integrated biomarker in the context of subclinical brain injury.

## 2 Materials and methods

### 2.1 Study population and study design

Three hundred twenty-seven consecutive patients without a history of cerebrovascular disease who presented to the neurology clinic and underwent cerebral magnetic resonance imaging (MRI) between January 2021 and May 2023 were analyzed retrospectively. All patients were outpatients who presented to the neurology clinic for non-specific neurological symptoms such as headache, dizziness, or screening for vascular risk. No inpatients were included.

Patients with a history of stroke (*n* = 11), atrial fibrillation/flutter (*n* = 16), moderate or severe cardiac valvular disease (*n* = 6), chronic or acute kidney failure (eGFR <30 mL/min/1.73 m^2^ or dialysis- dependent patients, acute kidney injury) (*n* = 6), advanced liver disease or cirrhosis (*n* = 3), active malignancy or patients undergoing chemotherapy/radiotherapy (*n* = 5), autoimmune or inflammatory disorders (e.g., systemic lupus erythematosus, rheumatoid arthritis) (*n* = 2), severe hematological disorders (e.g., significant anemia, coagulation disorders) (*n* = 2), severe cardiac disease, including recent myocardial infarction or heart failure with reduced ejection fraction (EF < 30%) (*n* = 2), neurological disorders such as dementia, epilepsy, or neurodegenerative diseases (*n* = 2) were excluded. After these exclusions, the study population consisted of 272 patients, with a median follow-up period of 37 months. The study’s primary outcomes included cerebrovascular events and atrial fibrillation/flutter, which were determined through national health insurance records and follow-up via trans-telephonic communication. Structured telephone interviews were conducted using a standardized questionnaire administered by trained personnel to ensure uniform outcome assessment. Cerebrovascular events were defined as ischemic or hemorrhagic strokes diagnosed by a physician and confirmed via neuroimaging (CT or MRI). Atrial fibrillation/flutter was determined based on ECG or Holter-confirmed diagnosis documented in national health insurance records or medical charts. Outcome data were obtained through centralized insurance records and corroborated during structured telephone follow-up using a standardized questionnaire. Baseline demographic characteristics and laboratory findings were obtained from the hospital’s electronic database. Blood samples for laboratory analyses were drawn at the time of hospital admission. Serum albumin and creatinine levels measured at admission were used to calculate the sACR. Due to limitations in data granularity during retrospective follow-up, cerebrovascular events and atrial fibrillation/flutter were analyzed as a composite outcome. Although atrial fibrillation/flutter is a risk factor rather than an outcome, it was included in the composite endpoint because of its shared thromboembolic mechanisms with stroke and its clinical relevance in silent cerebrovascular disease. This approach also improved event ascertainment in this retrospective design. This approach was chosen to enhance outcome ascertainment and reflect the shared pathophysiological basis of these conditions in silent cerebral infarction.

### 2.2 Statistical analysis

Data was analyzed using SPSS software version 21.0 (SPSS Inc., Chicago, IL, USA). The Kolmogorov–Smirnov test was employed to evaluate the normality of continuous variables. Descriptive statistics for normally distributed variables were reported as mean ± standard deviation, while non-normally distributed variables were presented as median (interquartile range). Categorical data were summarized as frequencies and percentages. Comparisons of non-normally distributed continuous variables between groups were conducted using the Mann–Whitney *U* test, whereas categorical variables were compared using Fisher’s exact test or Chi-square test. The Independent Samples *t*-test was used for normally distributed continuous variables. A *p*-value of <0.05 was considered statistically significant. Correlation analysis was performed using Pearson’s correlation coefficient for normally distributed variables and Spearman’s rank correlation coefficient for non-normally distributed variables. Specifically, Pearson and Spearman correlation analyses were used to evaluate the relationships between sACR and age, serum creatinine, and left atrial volume index (LAVI). Univariable and multivariable logistic regression analyses were employed to identify independent predictors of SCI. Variables included in the multivariable logistic regression model were age, left atrial volume index (LAVI), HDL-cholesterol, and sACR, selected based on clinical relevance and a univariable *p*-value threshold of <0.10. The optimal threshold for predicting SCI, with the highest sensitivity and specificity, was determined using receiver-operating characteristic (ROC) curve analysis. The Youden Index was applied to identify the cut-off point that maximized the sum of sensitivity and specificity. Time-to-event analyses for the occurrence of cerebrovascular events and atrial flutter/fibrillation were performed using the Kaplan–Meier method, and differences between groups were assessed with log-rank tests. Missing data were minimal (<2% for all variables) and were handled using pairwise deletion to ensure complete-case analysis without compromising statistical integrity.

### 2.3 Brain MRI

All brain MRIs were obtained at the time of the patients’ initial presentation to the neurology outpatient clinic, most commonly for non-specific symptoms such as headache, dizziness, or for high-risk screening purposes. Routine follow-up MRIs were not performed.

Patients who underwent brain MRI were included after applying the exclusion criteria. Cerebral imaging was performed using a 3 T Siemens MAGNETOM Skyra MRI system with a section thickness of 4 mm, FOV read 240 mm, FOV phase 93.8%, TR 9000 ms, and TE 81 ms. Silent cerebral infarctions (SCIs) were defined as lesions measuring between 3 mm and 15 mm in size, appearing hyperintense on T2 FLAIR sequences and hypointense on T1-weighted images, typically located in the deep white matter. Lesions smaller than 3 mm or larger than 15 mm and caps, pencil-thin lesions in the periventricular area, Virchow-Robin spaces, and perivascular spaces were excluded from SCI classification (18). Specifically, lesions exceeding 15 mm were not reclassified and were excluded from the analysis entirely. Although lesions >15 mm were excluded from SCI classification, subgroup analysis of these excluded lesions could not be performed, as they were not retained in the final dataset due to predefined exclusion criteria. All DW-MRI scans were independently reviewed by two interventional radiologists with expertise in stroke, who were blinded to the patient’s clinical information.

### 2.4 Ethical approval

This study was approved by the Başkent University Institutional Review Board (Project No: KA25/30) and was conducted following institutional ethical guidelines. Additionally, the research adhered to the principles outlined in the Declaration of Helsinki, ensuring the ethical conduct of medical research involving human subjects.

## 3 Results

The study included 272 patients with a mean age of 56.7 ± 7.9 years, of whom 72.1% were female. Missing data were minimal (<2% for all variables) and were handled using pairwise deletion to ensure complete-case analysis without compromising statistical integrity. Patients were stratified into two groups based on the presence of SCI. Table 1 summarizes both groups’ demographic, clinical, and laboratory characteristics. There were no significant differences between the groups regarding gender, diabetes mellitus, hypertension, dyslipidemia, smoking status, or body mass index (BMI). However, patients in the SCI group were significantly older (59.5 ± 6.6 vs. 54.7 ± 8.2, *p* < 0.001).

**Table 1:** Baseline characteristics of the patients according to the silent cerebral infarction.

Patient characteristics	SCI present	SCI absent	Total	*p*-value
	(*n* = 115)	(*n* = 157)	(*n* = 272)	
Age	59.5 ± 6.6	54.7 ± 8.2	56.7 ± 7.9	<0.001
Body mass index, kg/m^2^	32.2 ± 5.4	31.8 ± 4.6	32 ± 4.9	0.532
Body surface area, m^2^	1.86 ± 0.15	1.89 ± 0.18	1.88 ± 0.17	0.066
Gender (Female), *n* (%)	90 (78.3)	106 (67.5)	196 (72.1)	0.051
Smoking, *n* (%)	34 (29.6)	57 (36.3)	91 (33.5)	0.244
Diabetes mellitus, *n* (%)	18 (15.7)	26 (16.6)	44 (16.2)	0.841
Hypertension, *n* (%)	73 (63.5)	94 (59.9)	167 (61.4)	0.546
Dyslipidemia, *n* (%)	14 (12.2)	15 (9.6)	29 (10.7)	0.489
Chronic obstructive pulmonary disease, *n* (%)	18 (15.7)	23 (14.6)	41 (15.1)	0.819
Left ventricular ejection fraction, %	62.1 ± 2.8	62.3 ± 2.9	62.2 ± 2.8	0.505
LAVI, mL/m^2^	32.5 ± 5.4	30.4 ± 4.3	31.3 ± 4.8	<0.001
Fasting blood glucose, mg/dL	101 (75–205)	100 (76–294)	100.5 (75–294)	0.395
Blood urea nitrogen, mg/dL	18.6 ± 5.6	18.5 ± 5.9	18.6 ± 5.8	0.820
Creatinine, mg/dL	0.88 ± 0.17	0.78 ± 0.18	0.82 ± 0.18	<0.001
ALT, U/L	17 (7–53)	20 (8–199)	19 (7–199)	0.003
AST, U/L	17 (10–43)	20 (9–100)	19 (9–100)	0.009
Sodium, mmol/L	139.8 ± 2.6	140.3 ± 2.5	140 ± 2.5	0.115
Potassium, mEq/L	4.4 ± 0.4	4.3 ± 0.3	4.3 ± 0.4	0.098
Total cholesterol, mg/dl	200.8 ± 39.7	201.6 ± 44.4	201.3 ± 42.4	0.886
LDL-C, mg/dl	132 ± 32.8	127.4 ± 35.5	129.4 ± 34.4	0.276
HDL-C, mg/dl	42.8 ± 9.3	46.7 ± 11.1	45.1 ± 10.6	0.003
Triglyceride, mg/dl	183.6 ± 59	181.1 ± 75.9	182.1 ± 69.1	0.774
White blood cell, ×10^9^/L	7.20 ± 1.93	7.22 ± 1.90	7.21 ± 1.91	0.923
Hemoglobin, g/dl	13.8 ± 1.2	13.5 ± 1.5	13.6 ± 1.4	0.159
CRP, mg/dL	0.6 (0.3–5.6)	0.6 (0.3–9)	0.6 (0.3–9)	0.362
Albumin, g/dl	4.1 ± 0.3	4.2 ± 0.3	4.1 ± 0.3	0.506
sACR	4.8 (2.9–7)	5.6 (2.8–14)	5.1 (2.8–14)	<0.001
Outcome, *n* (%)	18 (15.7)	6 (3.8)	24 (8.8)	0.001
Follow-up, months	35 (5–42)	37 (16–43)	37 (5–43)	0.012

SCI, Silent cerebral infarction; LAVI, Left atrial volume index; ALT, Alanine transaminase; AST, Aspartate transaminase; LDL-C, Low-density lipoprotein cholesterol; HDL-C, High-density lipoprotein cholesterol; CRP, C-reactive protein; sACR, Serum albumin-to-creatinine ratio.

Regarding laboratory parameters, creatinine levels were significantly higher in the SCI group. In contrast, sACR, high-density lipoprotein cholesterol (HDL-C), alanine transaminase (ALT), and aspartate transaminase (AST) levels were significantly lower compared to the non-SCI group. Other laboratory parameters showed no significant differences between the groups. The echocardiographic evaluation revealed a significant difference in mean left atrial volume index (LAVI) values, with the SCI group exhibiting higher LAVI values (32.5 ± 5.4 vs. 30.4 ± 4.3, *p* < 0.001). Correlation analysis showed statistically significant but weak associations between sACR and age, serum creatinine, and LAVI (all |*r*| < 0.3). Patients were followed for a median duration of 37 months (interquartile range: 5–43 months). During the follow-up period, adverse outcomes occurred in 8.8% of the study population, with a significantly higher incidence in the SCI group compared to the non-SCI group [15.7% (*n* = 18) vs. 3.8% (*n* = 6), respectively]. Among the 24 patients with adverse outcomes, 15 experienced ischemic stroke, 3 had hemorrhagic stroke, and six developed atrial fibrillation or flutter. The shorter follow-up duration observed in the SCI group compared to the non-SCI group may be attributable to increased mortality among patients with SCI. The outcomes included physician-diagnosed ischemic or hemorrhagic stroke confirmed by imaging and new-onset or recurrent atrial fibrillation/flutter confirmed by ECG or Holter monitoring. Events were identified through national health insurance databases and structured telephone interviews. Patients were stratified into two groups—“Outcome present” and “Outcome absent”—based on the occurrence of either cerebrovascular events or atrial arrhythmias during follow-up. Table 2 compares these two groups’ demographic, clinical, and laboratory characteristics.

**Table 2:** Baseline characteristics of the patients according to the outcomes.

Patient characteristics	Outcome present (*n* = 24)	Outcome absent (*n* = 248)	Total (*n* = 272)	*p*-value
Age	60.6 ± 7.3	56.4 ± 8	56.7 ± 8	0.014
Body mass index, kg/m^2^	31.4 ± 5.3	32.1 ± 5	32 ± 5	0.535
Body surface area, m^2^	1.85 ± 0.17	1.88 ± 0.17	1.88 ± 0.17	0.351
Gender (Female), *n* (%)	17 (70.8)	179 (72.2)	196 (72.1)	0.889
Smoking, *n* (%)	11 (45.8)	80 (32.3)	91 (33.5)	0.178
Diabetes mellitus, *n* (%)	4 (16.7)	40 (16.1)	44 (16.2)	0.566
Hypertension, *n* (%)	10 (41.7)	157 (63.3)	167 (61.4)	0.058
Dyslipidemia, *n* (%)	8 (33.3)	21 (8.5)	29 (10.7)	<0.001
Chronic obstructive pulmonary disease, *n* (%)	6 (25)	35 (14.1)	41 (15.1)	0.132
Left ventricular ejection fraction, %	62 ± 2.7	62.2 ± 2.8	62.2 ± 2.8	0.735
LAVI, mL/m^2^	33.4 ± 8	31.1 ± 4.4	31.3 ± 4.8	0.156
Fasting blood glucose, mg/dL	102 (75–205)	100 (76–294)	100.5 (75–294)	0.570
Blood urea nitrogen, mg/dL	18.4 ± 5.2	18.6 ± 6	18.6 ± 5.8	0.876
Creatinine, mg/dL	0.88 ± 0.16	0.82 ± 0.18	0.82 ± 0.18	0.113
ALT, U/L	17 (8–35)	19 (7–199)	19 (7–199)	0.291
AST, U/L	17 (13–37)	19 (9–100)	19 (9–100)	0.154
Sodium, mmol/L	139.5 ± 2.5	140.1 ± 2.6	140 ± 2.5	0.304
Potassium, mEq/L	4.3 ± 0.3	4.3 ± 0.4	4.3 ± 0.3	0.977
Total cholesterol, mg/dl	209.1 ± 39.3	200.5 ± 42.6	201.3 ± 42.4	0.341
LDL-C, mg/dl	141.1 ± 28.5	128.3 ± 34.7	129.4 ± 34.4	0.080
HDL-C, mg/dl	46.3 ± 11.4	45 ± 10.5	45 ± 10.6	0.545
Triglyceride, mg/dl	185.8 ± 54.1	181.8 ± 70.5	182.2 ± 69.1	0.785
White blood cell, ×10^9^/L	7.4 ± 2	7.2 ± 2	7.2 ± 1.9	0.494
Hemoglobin, g/dl	13.8 ± 1.6	13.6 ± 1.4	13.6 ± 1.4	0.644
CRP, mg/dL	0.6 (0.4–2.9)	0.6 (0.3–9)	0.6 (0.3–9)	0.446
Albumin, g/dl	4.1 ± 0.2	4.2 ± 0.3	4.1 ± 0.3	0.637
sACR	4.5 (3.2–7)	5.2 (2.8–14)	5.1 (2.8–14)	0.062
Follow-up, months	17.5 (5–33)	37 (27–43)	37 (5–43)	<0.001
SCI, *n* (%)	18 (75)	97 (39.1)	115 (42.3)	0.001
Time to advers event, months (median [IQR])	13.2 [7.1–23.6]	—	—	—

LAVI, Left atrial volume index; ALT, Alanine transaminase; AST, Aspartate transaminase; LDL-C, Low-density lipoprotein cholesterol; HDL-C, High-density lipoprotein cholesterol; CRP, C-reactive protein; sACR, Serum albumin-to-creatinine ratio; SCI, Silent cerebral infarction.

All-cause mortality was not systematically collected and could not be directly analyzed in this study.

Older age was significantly associated with adverse outcomes (60.6 ± 7.3 vs. 56.4 ± 8, *p* = 0.014). No significant differences were observed between the outcome and non-outcome groups regarding BMI, body surface area, gender, smoking status, diabetes mellitus, hypertension, or chronic obstructive pulmonary disease, except for dyslipidemia and SCI. Dyslipidemia (33.3% vs. 8.5%, *p* < 0.001) and SCI (75% vs. 39.1%, *p* = 0.001) were significantly more prevalent in patients with adverse outcomes. In contrast, the two groups had no significant differences in laboratory parameters.

**Figure 1:**
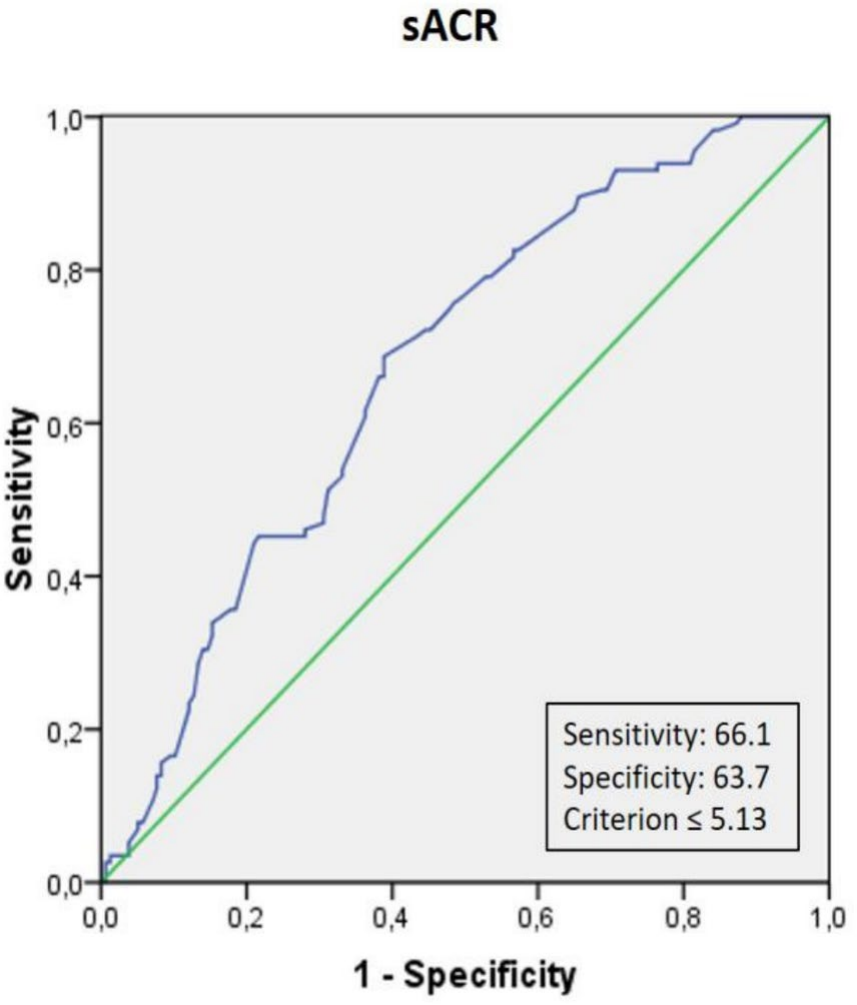
Receiver operating characteristic (ROC) curve analysis of serum albumin-to-creatinine ratio (sACR) for predicting silent cerebral infarction (SCI). The optimal cut-off value of sACR for predicting SCI was ≤5.13, with a sensitivity of 66.1% and a specificity of 63.7%.

Table 3 outlines the univariable and multivariable logistic regression analyses of factors associated with SCI, including age, LAVI, HDL-C, and sACR. After adjusting for confounders, each 1-point decrease in sACR was independently associated with a 1.790-fold increase in the likelihood of SCI (95% CI: 1.384–2.315, *p* < 0.001). Receiver-operating characteristic (ROC) curve analysis identified an sACR threshold of ≤ 5.13, providing the best sensitivity (66.1%) and specificity (63.7%) for predicting SCI (AUC: 0.674, 95% CI: 224 0.611–0.737, *p* < 0.001) (Figure 1). The distribution of follow-up durations across the co-hort is shown in Supplementary Figure 1. The interquartile range spans from 5 to 43 months, with a median of 37 months.

**Table 3:** Univariable and multivariable logistic regression analyses determine the independent predictors of silent cerebral infarction.

Univariable	*p*-value	OR	95% CI		Multivariable	*p*-value	OR	95% CI	
			Lower	Upper				Lower	Upper
Age	<0.001	0.920	0.889	0.952	Age	<0.001	0.921	0.886	0.958
LAVI	0.001	0.913	0.865	0.963	LAVI	0.009	0.923	0.868	0.980
HDL-C	0.003	1.038	1.013	1.064	HDL-C	0.009	1.038	1.009	1.068
sACR	<0.001	1.809	1.418	2.308	sACR	<0.001	1.790	1.384	2.315

LAVI, Left atrial volume index; HDL-C, High-density lipoprotein cholesterol; sACR, Serum albumin-to-creatinine ratio.

Although creatinine, ALT, and AST were initially considered, they were excluded from the multivariable model due to high collinearity with sACR and lack of consistent predictive value in univariable analysis. Including these variables would have introduced multicollinearity and potentially compromised model stability.

**Figure 2:**
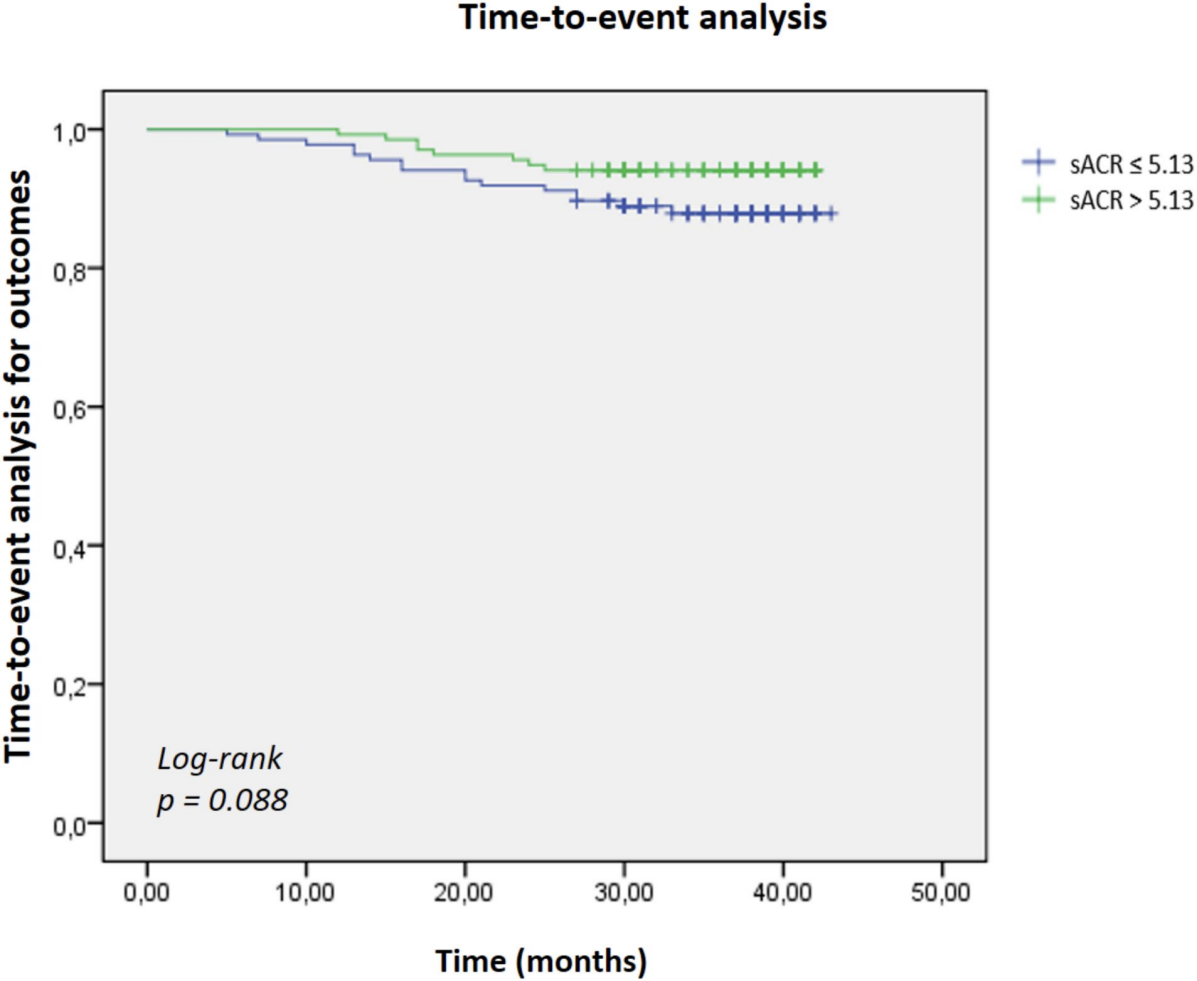
Kaplan–Meier survival analysis comparing cumulative survival rates between patients with low (≤5.13) and high (>5.13) serum albumin-to-creatinine ratio (sACR). Patients in the low sACR group exhibited lower survival rates over the follow-up period; however, this did not reach statistical significance (log-rank *p* = 0.088).

Patients were subsequently categorized into low and high sACR groups based on the cut-off value, and Kaplan–Meier survival analysis was conducted. The Kaplan–Meier analysis showed a trend toward higher outcome rates in the low sACR group; however, this did not reach statistical significance (log-rank *p* = 0.088) (Figure 2).

## 4 Discussion

Silent cerebral infarction (SCI) is a prevalent but often underrecognized cerebrovascular condition characterized by asymptomatic ischemic lesions detected through brain imaging. Despite their silent nature, these lesions are associated with an increased risk of future stroke, cognitive decline, and cardiovascular morbidity (19, 20). Understanding risk factors associated with SCI is essential for early identification and preventive strategies. This study focused on serum albumin to creatinine ratio (sACR) as a potential novel biomarker for predicting SCI and adverse outcomes.

While serum albumin-to-creatinine ratio (sACR) has been studied in the context of cardiovascular diseases such as myocardial infarction, heart failure, and sepsis (7–9), its association with cerebrovascular pathology, and specifically with silent cerebral infarction (SCI), has not been previously explored. In contrast, multiple studies have investigated the urinary albumin-to-creatinine ratio (uACR) with stroke risk and intracranial atherosclerosis (10, 11). These investigations have highlighted the role of albuminuria as a marker of microvascular damage, reinforcing the relevance of renal and vascular interplay in cerebrovascular disease. Our study extends this paradigm by focusing on sACR. This serum-based biomarker is routinely measured in clinical practice, offering a cost-effective and accessible alternative for assessing subclinical cerebrovascular vulnerability. To our knowledge, this is the first study to demonstrate a significant association between lower sACR levels and the presence of SCI, underscoring its potential utility in early risk stratification.

Numerous studies have shown an inverse relationship between serum albumin levels and the risk of various cardiovascular and cerebrovascular conditions in the general population, including coronary artery disease, heart failure, atrial fibrillation, stroke, and venous thromboembolism (20–25). As observed in preclinical studies, serum albumin has neuroprotective properties in acute stroke. It is a key regulator of oncotic pressure, counterbalancing hydrostatic blood pressure to maintain intravascular volume. Additionally, serum albumin exhibits significant antioxidant and anti-inflammatory effects while inhibiting platelet aggregation. These mechanisms collectively may help mitigate cerebral ischemia and edema during cerebral infarction, potentially leading to better clinical outcomes (26, 27).

These physiological roles contribute to maintaining endothelial integrity and vascular homeostasis. Therefore, the serum albumin-to-creatinine ratio (sACR) integrates nutritional status, renal function, and vascular health into a composite marker that may reflect an individual’s susceptibility to silent cerebrovascular injury. In our study, each 1-point decrease in sACR was associated with a 1.79-fold increase in the likelihood of SCI, supporting its potential role as a complementary biomarker in cerebrovascular risk stratification.

Previous studies have shown that SCIs are significantly more prevalent than symptomatic strokes, with estimates suggesting a fivefold higher incidence in the general population (28). Their prevalence increases with age and is generally higher in women. Among traditional cardiovascular risk factors, hypertension has been the most consistently associated with SCI, whereas other factors such as dyslipidemia, diabetes, and smoking show less consistent correlations (29–31). Interestingly, in our study, hypertension and other risk factors did not significantly correlate with SCI. This may be due to limitations in sample size or population characteristics, which underscores the need for a cautious interpretation of these negative findings. Notably, the SCI group exhibited higher creatinine levels and lower HDL-C levels, supporting the hypothesis that renal impairment and dyslipidemia may contribute to silent cerebrovascular damage. However, despite similar levels of total cholesterol, LDL-C, and triglycerides across groups, the prevalence of diagnosed dyslipidemia was higher in the SCI group. The higher prevalence of dyslipidemia despite similar laboratory lipid levels between groups may reflect effective lipid-lowering treatments, such as statins, which were not systematically recorded in this retrospective dataset. Specifically, numerous researchers have explored the potential link between renal dysfunction and SCI, given the structural and functional similarities of the microvascular networks in the kidney and brain (32). Mechanistically, renal dysfunction can promote a hypercoagulable state, increasing the risk of thromboembolic cerebral ischemia (33–35).

Renal dysfunction may contribute to endothelial injury through multiple mechanisms, including activation of the renin-angiotensin-aldosterone system, heightened inflammatory responses, and increased oxidative stress, all of which can exacerbate microvascular damage. This pathological vascular damage leads to lipohyalinosis in renal arterioles and endothelial dysfunction, processes analogous to those implicated in silent brain ischemia (36, 37). While both hypercoagulability and endothelial damage provide plausible explanations for the relationship between renal dysfunction and SCI, the causal direction of this association—whether renal dysfunction precipitates SCI or vice versa—remains uncertain. By integrating serum albumin, a marker of nutritional and anti-inflammatory status, with creatinine, an indicator of renal function, the serum albumin-to-creatinine ratio (sACR) provides a composite reflection of systemic vascular health. In our study, a lower sACR was independently associated with the presence of SCI, with each 1-point decrease in sACR corresponding to a 1.79-fold increase in SCI risk.

Additionally, Kaplan–Meier analysis demonstrated that patients with a low sACR (≤ 4.59) exhibited significantly higher rates of adverse outcomes—defined as incident stroke or atrial fibrillation/flutter—during follow-up compared to those with higher sACR levels (log-rank *p* = 0.009). Importantly, all-cause mortality was not included as an endpoint in this analysis. While these findings suggest that sACR may have prognostic utility in identifying individuals at increased risk for silent cerebrovascular injury and subsequent adverse events, they should be interpreted as hypothesis-generating. The causal relationship between sACR and SCI remains uncertain; it is unclear whether low sACR directly contributes to the development of silent infarctions or reflects underlying pathophysiological processes such as systemic inflammation, oxidative stress, and endothelial dysfunction. Prospective studies with larger sample sizes, mortality-specific endpoints, and longitudinal follow-up are warranted to validate these observations and elucidate the potential of sACR-guided risk stratification strategies in cerebrovascular disease.

Although cerebrovascular events and atrial fibrillation/flutter represent distinct clinical outcomes, they were analyzed as a composite endpoint in this study due to the retrospective design and the need for pragmatic event ascertainment. This approach was chosen to reflect the shared vascular and thromboembolic mechanisms underlying silent cerebral infarction and arrhythmogenesis, thereby enhancing the interpretability of adverse outcome data within the constraints of retrospective follow-up. Notably, our Kaplan–Meier analysis demonstrated significantly higher adverse outcome rates—comprising stroke and atrial fibrillation/flutter—in patients with low sACR levels. At the same time, all-cause mortality was not included as an endpoint in this analysis. Although the SCI group exhibited a shorter follow-up duration, which may suggest higher mortality, this remains speculative and could not be directly assessed within the scope of our dataset.

Given these limitations, our findings should be interpreted as hypothesis-generating. The observed associations between lower sACR levels, SCI presence, and subsequent adverse outcomes underscore the need for larger, prospective studies that incorporate mortality-specific endpoints and longitudinal follow-up to validate the prognostic utility of sACR. Furthermore, as this study is cross-sectional, causality cannot be inferred from the associations observed. The independent predictive value of sACR in cerebrovascular risk stratification should be confirmed through well-designed prospective cohort studies.

This study has several limitations that should be acknowledged. First, the retrospective design inherently limits the ability to establish causal relationships between sACR and silent cerebral infarction (SCI). Second, the study population was derived from a single center, which may affect the generalizability of the findings to broader populations. Third, while robust statistical adjustments were applied, residual confounding cannot be entirely excluded, particularly given the lack of systematic data on medication use, such as statins, which may have influenced lipid profiles and vascular risk. Additionally, intracranial and extracranial vascular imaging was not routinely performed, precluding a comprehensive assessment of vascular pathology in relation to SCI. Importantly, all-cause mortality was not predefined as a study endpoint and could not be directly analyzed, limiting the evaluation of sACR’s prognostic value regarding survival outcomes.

Future prospective studies with larger, more diverse cohorts are necessary to validate the prognostic utility of sACR in cerebrovascular risk stratification. These studies should incorporate systematic documentation of clinical treatments, direct assessments of vascular morphology, cognitive outcomes, and mortality endpoints to comprehensively evaluate the clinical significance of sACR in both subclinical and overt cerebrovascular disease.

## 5 Conclusion

In conclusion, our study suggests that the serum albumin-to-creatinine ratio (sACR) may serve as a novel and accessible biomarker for identifying individuals at increased risk of silent cerebral infarction (SCI) and subsequent adverse cerebrovascular events. Lower sACR levels were independently associated with SCI presence and higher rates of stroke or atrial fibrillation/flutter during follow-up. Given its ease of measurement and cost-effectiveness, sACR holds potential as a complementary tool in cerebrovascular risk stratification. However, these findings should be interpreted as hypothesis-generating, and the prognostic significance of sACR requires validation in prospective, longitudinal studies that incorporate mortality endpoints and systematic assessments of vascular pathology.

## Data Availability

The original contributions presented in the study are included in the article/Supplementary material, further inquiries can be directed to the corresponding author.
