# Supplementary material for: Serum albumin-to-creatinine ratio as a novel and cost-effective biomarker for silent cerebral infarction: a retrospective cohort study
Source: Front Neurol. 2025 Aug 22;16:1633402. doi: 10.3389/fneur.2025.1633402 (PMC12411197; doi:10.3389/fneur.2025.1633402)
Supplement: Supplementary file 1 [file Image_1.pdf]

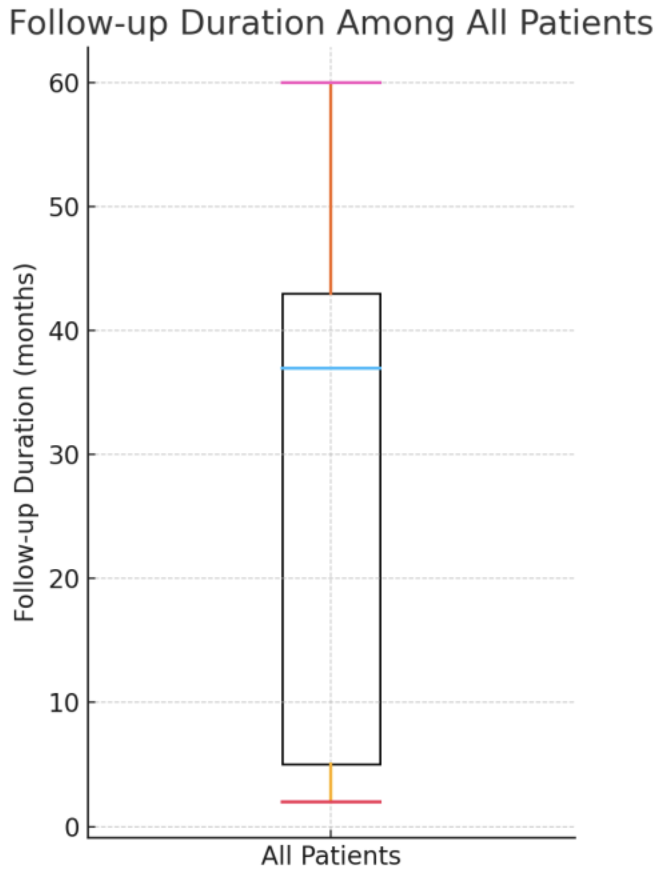

Supplementary Figure 1.
Boxplot showing the distribution of follow-up durations among all patients. The box spans the interquartile range (Q1 = 5 months to Q3 = 43 months), the thick line indicates the median (37 months), and the whiskers extend from the minimum 2 months) to the maximum (60 months).
